# Predictive Analytics for Retention in Care in an Urban HIV Clinic

**DOI:** 10.1038/s41598-020-62729-x

**Published:** 2020-04-14

**Authors:** Arthi Ramachandran, Avishek Kumar, Hannes Koenig, Adolfo De Unanue, Christina Sung, Joe Walsh, John Schneider, Rayid Ghani, Jessica P. Ridgway

**Affiliations:** 1 Center for Data Science and Public Policy, Department of Computer Science, University of Chicago, Chicago, United States; 2 Chicago Center for HIV Elimination, Department of Medicine, University of Chicago, Chicago, United States

**Keywords:** HIV infections, Epidemiology, Risk factors

## Abstract

Consistent medical care among people living with HIV is essential for both individual and public health. HIV-positive individuals who are ‘retained in care’ are more likely to be prescribed antiretroviral medication and achieve HIV viral suppression, effectively eliminating the risk of transmitting HIV to others. However, in the United States, less than half of HIV-positive individuals are retained in care. Interventions to improve retention in care are resource intensive, and there is currently no systematic way to identify patients at risk for falling out of care who would benefit from these interventions. We developed a machine learning model to identify patients at risk for dropping out of care in an urban HIV care clinic using electronic medical records and geospatial data. The machine learning model has a mean positive predictive value of 34.6% [SD: 0.15] for flagging the top 10% highest risk patients as needing interventions, performing better than the previous state-of-the-art logistic regression model (PPV of 17% [SD: 0.06]) and the baseline rate of 11.1% [SD: 0.02]. Machine learning methods can improve the prediction ability in HIV care clinics to proactively identify patients at risk for not returning to medical care.

## Introduction

Consistent medical care is essential for the health of people living with HIV. HIV-positive individuals who receive regular medical care are more likely to receive antiretroviral therapy, less likely to develop Acquired Immune Deficiency Syndrome (AIDS), and have improved survival rates compared to HIV-positive individuals who do not receive regular medical care^1–3^. In the field of HIV medicine, patients who receive regular medical care are considered ‘retained in care.’ Retention in care is not only important for the individual health of people living with HIV, but also for public health. HIV-positive individuals who are retained in care and taking antiretroviral therapy are able to suppress the HIV viral level in their serum to undetectable levels, effectively eliminating the risk of transmitting HIV to others. Accordingly, retention in care is a critical pillar of public health agency plans to eliminate HIV transmission in the United States^4–6^.

Despite the clear benefits of retention in care for individual and public health, less than half of individuals living with HIV in the U.S. are retained in care. Lack of access to healthcare is one reason that patients may not be retained in care^7^. However, for patients who lack health insurance, state and federal programs such as the Ryan White HIV/AIDS Program provide funding to pay for HIV care visits and antiretroviral medications. Despite these programs, many patients living with HIV still do not regularly attend medical appointments. Additional barriers to retention in care remain, including mental illness, substance use, insecure housing, poverty, neighborhood violence, and stigma^8–16^.

Interventions that are effective for improving retention in care include intensive case management, peer navigation, and multi-faceted outreach programs^17–25^. These interventions are resource intensive and difficult to provide for all patients in limited resource settings. Furthermore, not all patients are at risk for retention failure nor would benefit from intensive retention interventions. Therefore, methods are needed to identify and prioritize HIV-positive patients at highest risk for falling out of medical care.

Existing work on this problem has focused on two aspects: (1) using retrospective analysis to identify population level subgroups at risk for dropping out of care, such as African-American men who have sex with other men^26^, and (2) understanding root causes and barriers to retention in care. These approaches may be useful in describing vulnerability to falling out of care, but are less useful in proactively targeting retention resources. Prioritizing interventions using group level risk factors (e.g., men who have sex with men) can waste already scarce resources because it presumes that all members have uniform risk, neglecting individual circumstances and behaviors. In contrast, a more fine-grained machine learning approach to identify individuals at risk for falling out of care can overcome these shortcomings by building models tailored to individual features, rather than just group characteristics.

Machine learning methods are particularly well suited for early warning systems that inform interventions for patient retention because they (1) are optimized for future predictive accuracy, (2) can detect non-linear complex interactions (as opposed to traditional methods), (3) are able to rank and prioritize individuals according to risk score rather than group risk, and (4) combine multi-source data at different levels of granularity. Traditional methods (e.g., differential equation modeling or agent-based modeling) focus on understanding HIV transmission in aggregate rather than at the individual level and are not optimized for prediction. Accordingly, the aim of this study was to develop a machine learning predictive model of retention in HIV care among individuals in an urban HIV care clinic using electronic medical record (EMR) data, geospatial data, and US Census data. Our machine learning models are scalable, adaptive, and produce patient-level dynamic predictions.

## Methods

### Study sample

HIV-positive individuals 18 years of age and older who attended at least one medical appointment at the University of Chicago adult HIV care clinic between January 1, 2008 and May 31, 2015 were included in the study. The University of Chicago adult HIV care clinic is located on the south side of Chicago, a major U.S. HIV epicenter^27^. For all eligible patients, the following data were collected from the EMR: demographics, insurance information, other medical conditions, medications, HIV care provider, substance use history, and laboratory test results. Appointment attendance history including attended, cancelled, and missed visits was collected from the beginning of the study period up to one year after study enrollment (through May 31, 2016). Both billing diagnoses as well as clinician-assigned diagnoses documented in the “problem list” section of the EMR were collected. All medical encounters within the University of Chicago were collected including outpatient appointments in the HIV clinic, all other outpatient appointments, hospitalizations, and Emergency Department visits. Laboratory test results collected included HIV viral load, lymphocyte subset data (e.g., CD4 count), sexually transmitted infection (STI) test results, and toxicology test results. Patients’ addresses were geocoded and the travel distance and travel time to the clinic as well as the crime rate along the travel rate were calculated. Geocoding methods have been previously described^28^. Using data from the American Community Survey (US Census Bureau), characteristics of a patient’s community at the census tract level including racial composition, fraction of population on Supplemental Nutrition Assistance Program, commute characteristics and education levels were collected^29^. Patients were censored, meaning the machine learning system no longer generated a prediction for the patient for a given window of time, when they transferred care to another clinic or died.

### Predictor variables

Using the data described above, we generated a set of ~ 800 predictor variables (features) to be considered for inclusion in the machine learning models. Prior literature was used to guide feature creation, including factors previously shown to be associated with retention in HIV care, such as age, CD4 count, substance use, psychiatric illness, and prior visits^8–17^. Categories of features included demographics, diagnoses, location-based features, laboratory test results, medical visits, and specific providers seen. For each feature, measures were aggregated by time (e.g., count for the past six months, standard deviation for the past year, etc.) or time and space (e.g., the number of thefts in the patient’s residential census tract in the past six months). We explored a range of values for the time (6 months, 1 years, 3 years, all history) and space (by zipcode and census tract) aggregations as well as different aggregation functions (mean, minimum, maximum, standard deviation). Categorical variables (such as race) were dummified. We detail this list in the appendix (Appendix eTable 1).

This methodology allows the machine learning model to use the time and space aggregation of the feature that is most predictive of the final outcome. For example, if more recent (6 month) viral loads are better correlated with retention in care than viral loads from several years ago, the method will use the average viral load in the past six months rather than average viral load for the past three years.

### Missing data

Features with missing data had values imputed with the choice of value depending on the variable (*e.g*., a missing birth date resulted in an age assignment of the mean age of the population). For more details, see appendix (eTable 1). We also included a flag for whether or not the value was imputed as an additional predictor variable, allowing the model to use the missingness of a predictor variable as a predictor itself.

### Study outcomes

Two outcomes were studied: (1) *retention in care* and (2) *access to care*. Retention in care was defined as attending at least 2 HIV care visits greater than 90 days apart within a 12-month period^30^. This definition of *retention* is from the Health Resources and Services Administration HIV/AIDS Bureau (HRSA HAB). While there is no true “gold standard” of retention in care, this definition has been shown to be correlated with patient health outcomes including HIV viral suppression^31^. *Access to care* (also referred to as a 6 month gap) is defined as having a single HIV care visit within a 6 month period^31,32^. This metric is used by public health departments for the purposes of surveillance^27^. The outcome was predicted at the time of each patient’s HIV care appointment, replicating the workflow (and data available) in the clinic, in which the patient arrives for their appointment and then receives a risk score. This predicted risk score can then inform and prioritize interventions to improve future retention in care.

### Model training, validation, and selection

We tested the performance of 5 machine learning models in comparison to the current methods used by HIV clinicians for predicting retention and access to care. Methods comprised of regularized logistic regressions (l1 and l2), gradient boosting decision trees, decision trees, extra trees, and random forests. The five machine learning models were chosen to cover a large spectrum of possible classifiers and the spectrum of linear, tree, tree ensemble, and boosting models. Using Triage^33^, ~100 hyperparameter combinations for each model were tested, then fit to each training set^34^. Validation was performed using temporal cross validation^35^. Temporal cross validation was used instead of k-fold cross-validation to account for serial correlation and temporal patterns in the data and correctly replicate the modeling workflow in deployment. The data were divided into sets of model building cohorts and validation cohorts (alternatively, training set and test set), each of which is split by time (eFig. 1). This allows models to be developed on all appointments occurring before the year of prediction and tested on appointments occurring during the year of prediction. Model reporting complies with the Transparent Reporting of a Multivariable Prediction Model for Individual Prognosis or Diagnosis (TRIPOD) reporting guidelines^36^.

Model performance was evaluated using the positive predictive value (PPV) with a population threshold of 10% (i.e., appointments were ranked by their scores and the top 10% of those were classified as high risk of retention failure). The PPV is the percentage of individuals correctly identified by the model as at risk that go on to drop out of care (i.e., the number of true positives divided by the number of predicted positives). In order to use retention resources efficiently, the system should minimize false positives, which would minimize wasting resources on patients who would not drop out of care. The choice of threshold was driven by the authors’ clinic’s capacity for intervention – 10% of the population is approximately 150 appointments a year. We chose not to use Area Under the Curve (AUC), which is often reported for predictive models, because it is not appropriate for our limited resources setting as it captures the overall performance across every threshold. To prioritize a small number of individuals for intervention, positive predictive value ensures the model selected will minimize false positives within the intervention set.

For each model type, we chose the hyperparameters that consistently had high performance for each of the validation sets (*i.e*., each of the time periods). Specifically, we chose the model that most frequently was within 5% of the PPV of the best possible model over each time period (*e.g*., if the best possible PPV for a time period was 0.80, all models above 0.75 PPV were selected). This ensures that the final model selected is one that is both stable and has high performance.

### Performance evaluation

Predictions were made at the appointment level to simulate deployment in a clinical setting. For any prediction at the time of appointment, the training data and predictor variables included only information known before that point in time. We compared the PPV at 10% of the machine learning models to a logistic regression model based on the literature-identified features^37^, referred to as the ‘previous-state-of-the-art’ model. This ‘previous-state-of-the-art’ model uses the factors that clinicians might use to predict whether a patient will be retained in care based on previously published literature^37,38^. These features included demographics, age, race, gender, diagnosis of psychiatric illness, substance use history, viral load, and time since HIV diagnosis. We also compare our model results with the prior (the fraction of individuals who are not retained in care or not accessing care).

### Bias evaluation

Machine learning models deployed in this setting with many at-risk groups involved have the potential to disproportionately affect some sub-groups and exacerbate disparities. We audited our models using Aequitas^39^ to ensure that prediction errors do not disproportionately impact certain protected classes (e.g., racial minorities).

While bias can be measured in many ways, we focus on metrics that measure disproportionate false negatives since failing to detect people at risk for retention failure is presumably more harmful than detecting false positives in these groups. A patient at risk for retention failure who does not receive an intervention loses opportunities for underlying challenges to be addressed (*e.g*., transportation might be a challenge and a case worker might be able to help navigate public transit). On a group level, a group can be negatively impacted if they systematically do not receive an intervention when it is needed. To measure this impact, we use False Omission Rate (FOR), which is defined as the number of false negatives divided by the number of negative predictions (alternatively Negative Predictive Value). Identifying a patient to be falsely at risk carries less negative impact to the patient, though the clinic intervention can become more inefficient when the clinic staff intervene on patients who are falsely identified as high risk.

Given the racial composition of the patient population, we focused our attention on auditing models for parity in FOR by race. Specifically, we considered a model to be disparate if its FOR ratio of Black vs White is either less than 0.9 or greater than 1.1.

### Implementation

The system was built using Triage, an open source machine learning tool^33^, for building features, running models across a large hyperparameter space, model selection, and model evaluation. The data and results are stored in a PostGreSQL database. We used Python’s scikit-learn package for the machine learning models. The configuration file used to specify features and models can be found on GitHub^34^.

### Ethical review of study and waiver of consent

This study was approved by the University of Chicago Institutional Review Board (IRB). The IRB waived the need for informed consent as part of the study approval. Research was carried out in accordance with the ethical standards in the Declaration of Helsinki.

## Results

Over the study period, 713 patients attended at least one HIV care appointment (Table 1), accounting for 11,445 total visits. Of these appointments, between 8–12% of appointments were not followed by a subsequent appointment at least 90 days later within a 12-month period, indicating a lack of retention in care for that time period (eFig. 2). Also, of these appointments, approximately 10% of the appointments did not have a subsequent appointment in a six-month period (access to care).

**Table 1:** Characteristics of Study Population of 713 University of Chicago HIV Clinic Patients from January 1, 2008 through May 31, 2015.

Characteristics	Value
Age at first visit in study period, Mean (SD)	47.3 (13.6)
Female, No. (%)	314 (44%)
**Race**	
African American, No. (%)	585 (82%)
White, No. (%)	93 (13%)
Other, No. (%)	35 (5%)
**Insurance**	
Private, No. (%)	312 (44%)
Medicaid, No. (%)	309 (43%)
Medicare, No. (%)	85 (12%)
Number of attended appointments, Mean (SD)	19.5 (17)
**Census-based Aggregates**	
Fraction in zipcode that are African American, Mean (SD)	0.81 (0.29)
Fraction in zipcode with less than $10 k income, Mean (SD)	0.07 (0.04)
Fraction in zipcode with income between $10 k and $15 k, Mean (SD)	0.03 (0.02)
Fraction in zipcode on SNAP, Mean (SD)	0.14 (0.08)
Fraction in zipcode with high school education, Mean (SD)	0.38 (0.14)
Fraction in zipcode with some college, Mean (SD)	0.19 (0.06)
Fraction in zipcode with bachelors, Mean (SD)	0.13 (0.09)
**GIS measures**	
Distance from residence to clinic (miles), Mean (SD)	4.88 (3.65)
Travel time in minutes (public transit) from residence to clinic, Mean (SD)	43.5 (19.4)
Travel time in minutes (car) from residence to clinic, Mean (SD)	18.8 (10.7)
Average crime rate on route from from residence to clinic, Mean (SD)	0.11 (0.03)

### Model evaluation

#### Retention in care

**Figure 1:**
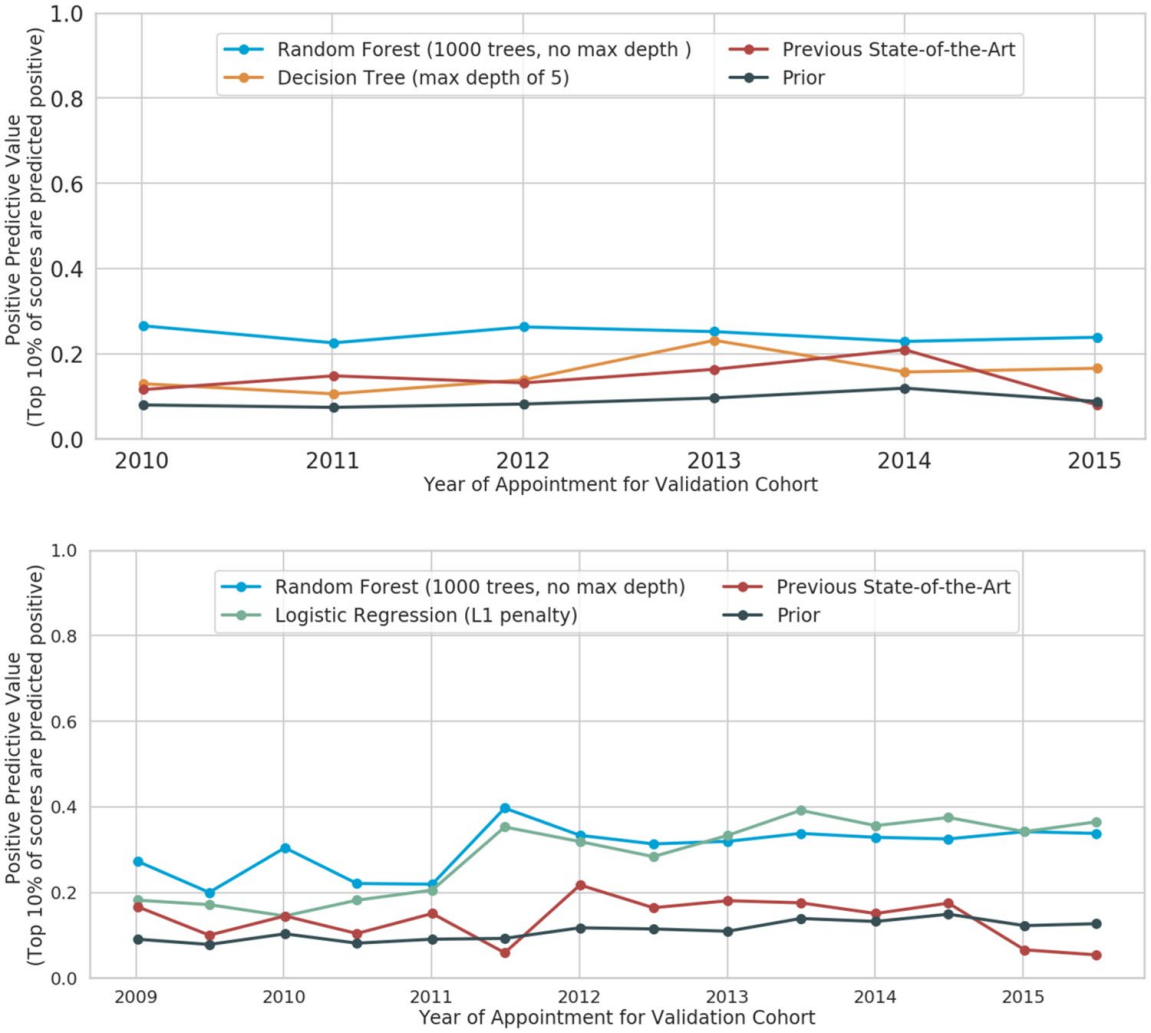
Positive Predictive Value of highest 10% risk scores for Retention in Care (top) and Access to Care (bottom) across model space: Positive Predictive Value (PPV) measures how many appointments were correctly predicted to have no follow-up (as defined by the HRSA HAB definition of retention) among the top 10% of appointments. The 10% threshold was chosen to match the resources the clinic has for launching an intensive intervention. The machine learning models shown below are the best performing model (blue) and the best performing model of an alternate model type (for retention in care, a decision tree, and for access to care in 6 months, a logistic regression).

The previous-state-of-the-art model had an average PPV of 14.1% [SD: 0.04] throughout the study period for the top 10% of predicted risk individuals, an improvement of 100% compared to the prior. The best performing models from each class of models had similar performance (Fig. 1 (top)). The best performing model was a random forest with 1000 estimators, maximum tree depth of 5, each leaf node having at least 2.5% of the samples, and each tree split requiring at least 10 samples. This model had a 200% improvement over the prior of 8–12% and 100% improvement over the previous-state-of-the-art model (PPV of 24.5% [SD: 0.01] for the top 10%). A simple decision tree had a lower performance with a PPV of 15.5% [SD: 0.04].

#### Access to care

The best performing model for access to care was a random forest with 1000 estimators, no specified maximum tree depth, each leaf node having at least 2.5% of the samples, and each tree split requiring at least 10 samples. This model had an average PPV of 34.6% [SD: 0.15] throughout the study period for the top 10% of appointments, a 300% improvement over the prior and 200% over the previous-state-of-the-art model (PPV of 17% [SD: 0.06]) (Fig. 1 (bottom)). This corresponds to approximately 50 additional appointments that are flagged as high risk of not having a follow-up appointment compared with the previous state of the art model.

While we focus on PPV at 10%, the chosen model can also be used to support interventions on a larger fraction of the population. eFigure 3 shows the change in PPV and sensitivity at different levels of intervention.

### Key predictor variables

**Figure 2:**
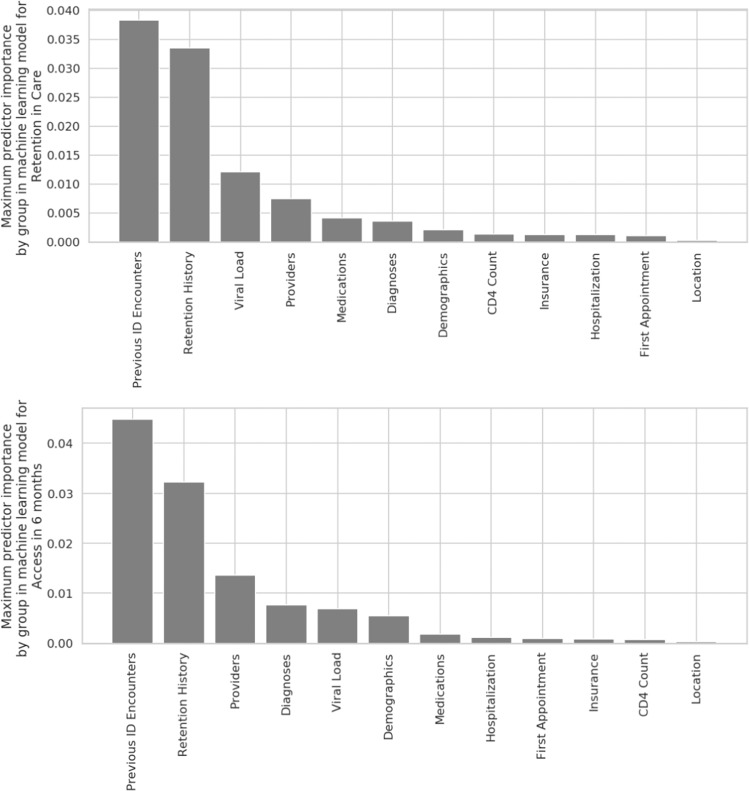
Features Learned by Machine Learning Models for (top) Retention in Care and (bottom) Access to Care: The feature importance of the random forest is the mean of the gain in purity of each of the underlying decision trees and is similar to logistic regression coefficients. The maximum importance within each class of predictor variables shows that the most important predictors for the model are based on the history of retention and the previous infectious disease clinic visits.

The models for both retention and access to care rely on similar predictor variables, sharing 80% of the top 20 predictors. A patient’s history of past retention in care and previous HIV care encounters are important predictors for the machine learning models for both retention in care and access to care in 6 months (Fig. 2). In general, the previous-state-of-the art model found demographic features important. The top features of the previous-state-of-the art model were demographic features such as race, ethnicity as well as features such as days since first appointment and number of days since diagnosis. The best Random Forest model initially found these features predictive, but as the system collected more data the Random Forest model found the medical history of a patient–retention history, appointments, history of lab tests–to be more predictive.

### Bias evaluation

**Figure 3:**
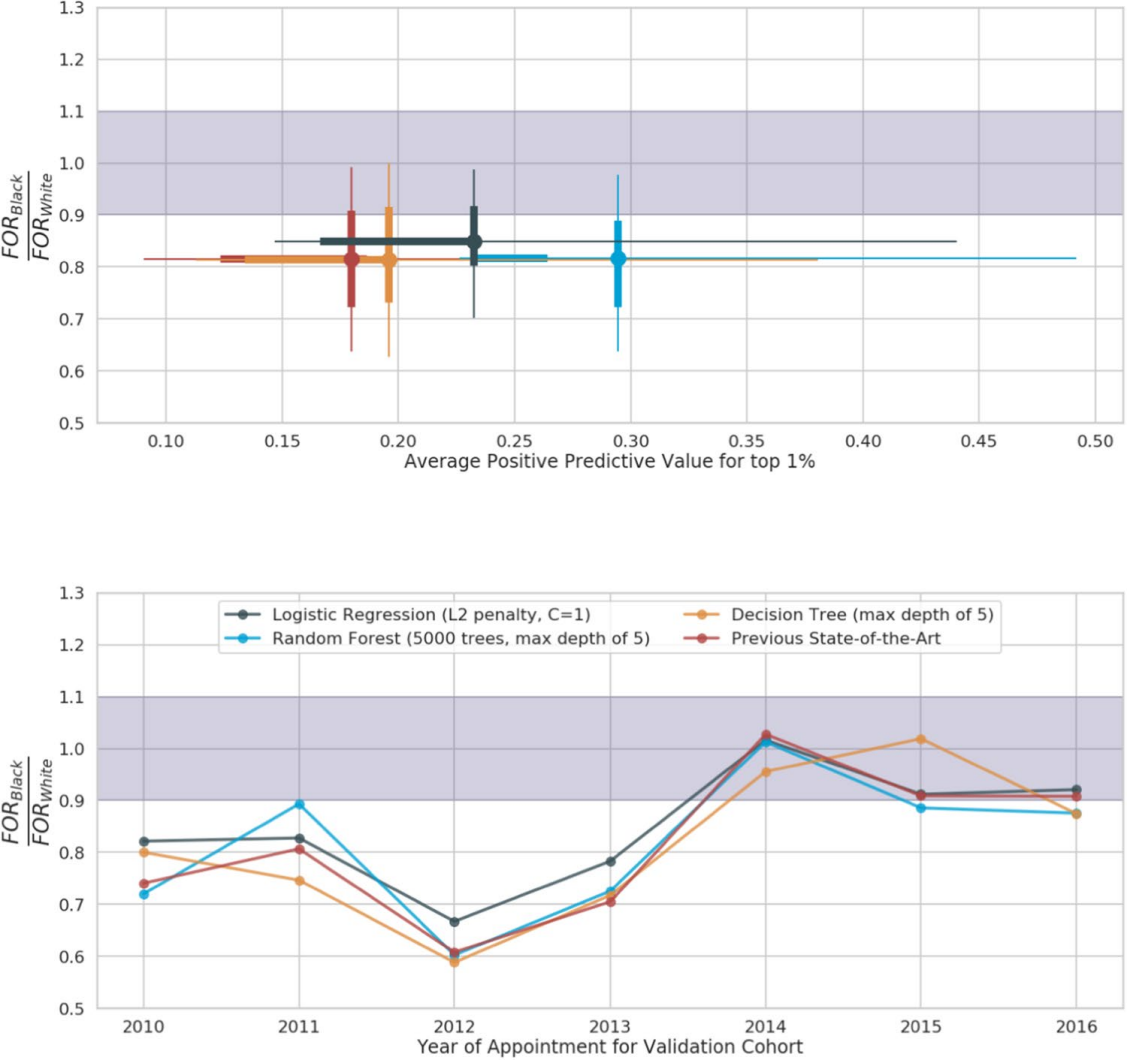
Trade off of performance vs fairness in models for retention in care: (top) There is a trade off in choosing models with high performance (x-axis) and minimal bias (y-axis). The circles show the average PPV and FOR. The lines show distribution of both PPV and FOR ratio over the different time periods. The thick lines show the first and third quartiles; the thin lines show the 5% and 95% percentiles. The purple band is the band of minimal disparity in FOR i.e., the ratio of the FOR for Black vs White races is within [0.9, 1.1] (bottom). Over time, the disparity in FOR for both our best performing machine learning models reduces. The machine learning model that is selected for best stable performance (blue) is better performing than the previous state of the art model (red). The best decision tree model (orange) has slightly lower performance and similar FOR ratios. The remaining models (black) were chosen for minimal disparity.

**Figure 4:**
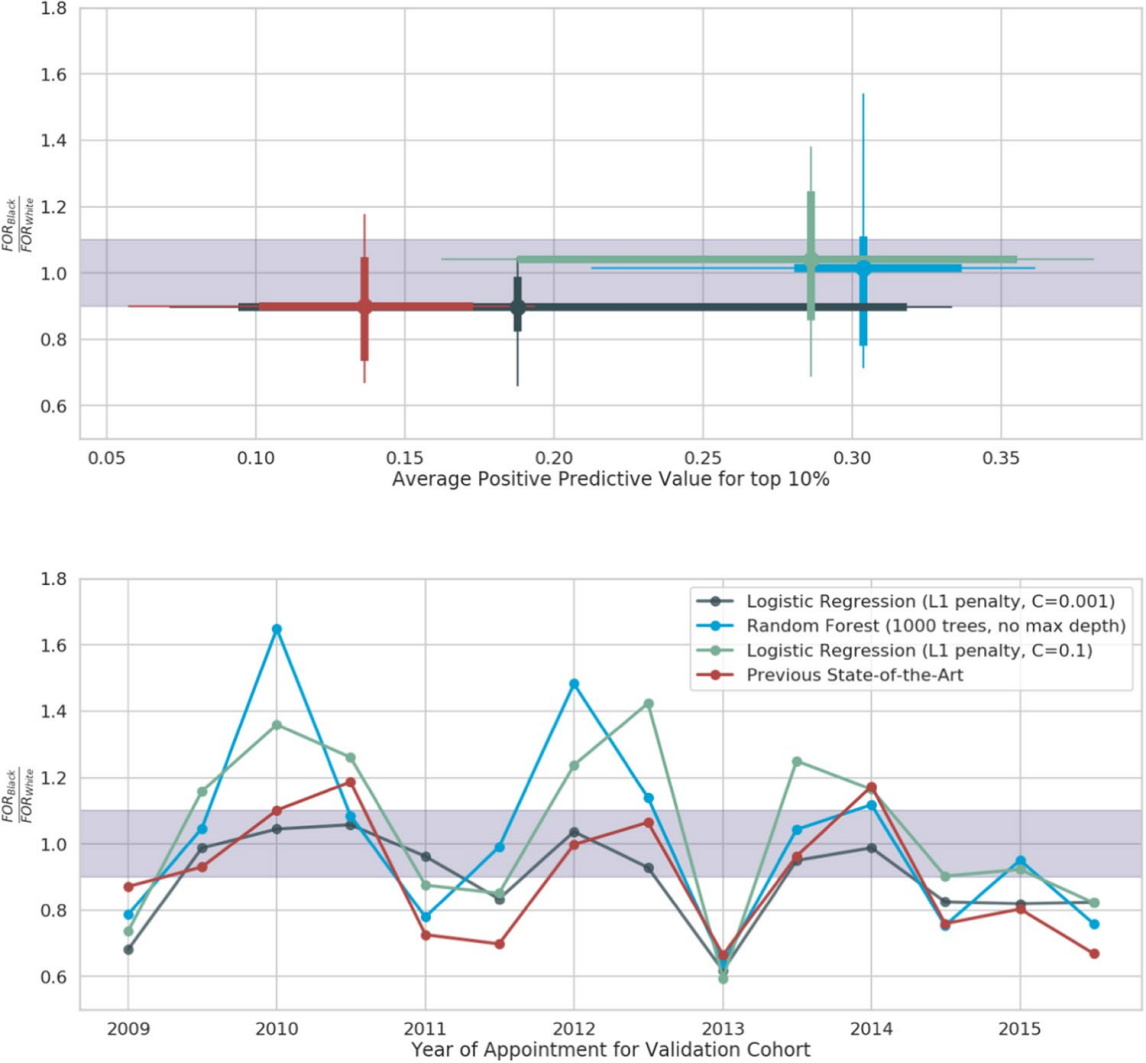
Trade off of performance vs fairness in models for accessing care: (top) There is a trade off in choosing models with high performance (x-axis) and minimal bias (y-axis). The circles show the average PPV and FOR. The lines show distribution of both PPV and FOR ratio over the different time periods. The thick lines show the first and third quartiles; the thin lines show the 5% and 95% percentiles. The purple band is the band of minimal disparity in FOR i.e., the ratio of the FOR for Black vs White races is within [0.9, 1.1]. Note that the x-axis goes from 0 to 0.4 to highlight the performance of the models. (bottom) Over time, the disparity in FOR for both our best performing machine learning models reduces. The machine learning model that is selected for best stable performance (blue) is better performing than the previous state of the art model (red). The best logistic regression model (green) has slightly lower performance and similar FOR ratios. The remaining models (black) were chosen for minimal disparity.

The machine learning model for retention in care had FOR 0.26 [SD 0.16] for black patients compared to 0.31 [SD 0.17] for white patients (Fig. 3). The previous-state-of-the-art model had FOR of 0.27 [SD 0.17] and 0.32 [SD 0.17] for black and white patients respectively. The machine learning model for access to care in six months had FOR 0.24 [SD 0.04] for black patients compared to 0.25 [SD 0.08] for white patients (Fig. 4). The previous-state-of-the-art model had FOR of 0.26 [SD 0.05] and 0.29 [SD 0.08] for black and white patients respectively. When selecting for models with minimal overall FOR disparity, there is a tradeoff – the average PPV of the lower disparity models is 18% and 22% lower for retention in care and accessing care respectively. The FOR ratios are calculated over a relatively small sample. The predicted positive group is approximately 120 appointments per year which are split into different racial categories. As a result, this metric is susceptible to variation from small population size.

## Discussion

This study demonstrates the potential of machine learning models to identify individual patients at the highest risk for falling out of HIV care, allowing busy HIV care clinics to direct limited resources toward patients who need them the most. To our knowledge, this is the first use of machine learning to understand retention in care among individuals living with HIV. Clinicians have difficulty predicting patients’ risk for missing appointments, and may be subject to bias in determining which patients would benefit from resource-intensive retention interventions^40^. Our machine-learning model had a higher PPV and was less biased than the previous-state-of-the-art logistic regression model.

Furthermore, while most prior literature regarding retention in care examines factors associated with retention at a single point in time, our model dynamically predicts retention longitudinally. Patients’ appointment attendance patterns change over time, with patients often transitioning in and out of care^41^. The method we developed provides a retention risk score at the visit level and recalculates the score at each subsequent visit, incorporating new data that becomes available as well as characteristics that change over time (e.g., prior appointment attendance, HIV viral load, substance use patterns, change of address, etc.).

We modeled two different definitions of healthcare utilization: retention in care and access to care. Both definitions are used in practice and described in the literature. Overall, the machine learning model for access to care had a greater performance improvement over the previous-state-of-the-art model compared to the model for retention in care. Therefore, the model for access to care may be more efficient to implement in practice for the same amount of intervention resources. This will have to be decided upon based on a clinic’s priorities for intervention.

We found that the most important predictor variables in the machine learning models for both retention and access were based on previous retention history and clinic visit history (e.g., total number of attended appointments). This is in keeping with prior literature that has shown that patients’ history of missing appointments is predictive of future missed appointments. Pence *et al*. reported that the most important predictor of future missed visits among HIV-positive patients is prior missed visits^42^. Other studies have found that low initial CD4 count and elevated HIV viral load are risk factors for poor retention^11,13^. We found that the existence of CD4 or viral load tests acts as a proxy for the existence of an appointment and is thus more relevant to retention than the exact values of the laboratory tests.

Other factors that have been reported in prior literature to be related to retention including race and age did not figure prominently in our model. However, these were important predictors in models built on earlier time periods, indicating that when other historical information is not available, these factors can be useful predictors. Additionally, our population was 82% African American and with a mean age of 48 years. We may not have had sufficient numbers of other races or young patients for these factors to influence retention outcomes in our model. Of note, geospatial factors including travel time to clinic, neighborhood crime rate, and neighborhood characteristics were not among the most important predictive features in the models. This may be because many of our patients live in neighborhoods with similar characteristics (i.e., high poverty, similar crime rates) on the south side of Chicago. When our methodology is applied to a different and more socioeconomically diverse patient population, these features may rank higher in importance. To our knowledge, this is the first use of bias auditing of predictive models in an HIV care setting. Further work is needed to understand how to mitigate the risk of exacerbating disparities.

Our study has several limitations. EMR data regarding patients’ diagnoses, medications, etc. may be inaccurate if providers do not accurately document and update patient data at each visit. Prior studies have shown wide variability in accuracy of billing diagnoses and incomplete problem list documentation in the EMR^43,44^. We attempted to limit inaccuracy due to poor documentation by incorporating multiple fields from the EMR. For example, patients with a history of substance abuse were detected not only by examining billing diagnoses for substance abuse, but also by collecting clinician-assigned diagnoses in the problem list, social history documentation of substance abuse, and toxicology screen results. Additionally, our EMR database only stores each patient’s most recent home address. Therefore, we were unable to account for changes in patients’ home address or living situations in our geospatial analyses. Furthermore, certain factors that may have an important impact on retention in care may not be captured within structured fields of the EMR, i.e., life stressors, social support, child care or other responsibilities, etc. In the future, we plan to incorporate natural language processing of unstructured clinical notes into our model to detect these factors.

Other sites can replicate the process presented here for extracting electronic data and incorporating them into machine learning systems using the in-house framework^33^ and our open source code. The vast majority of outpatient medical practices in the U.S. utilize EMRs^45^, allowing them to replicate our process. Our open source code is available at https://github.com/dssg/hiv-retention-public.

In summary, we have created a machine learning system to predict which patients are most likely not to be retained-in-care that creates a longitudinal and panoramic view of the patient, incorporating different types of data at different levels of granularity, that outperforms the previous-state-of-the-art model as well as being more adaptable, scalable, and fair. Future areas of study include incorporating the model into the EMR to allow it to be used in real time to direct retention resources for patients most at risk for falling out of care.

## Conclusions

Retention in care is crucial for individual and public health, yet the majority of people living with HIV in the United States are not retained in care. This study demonstrates that a machine learning framework to derive an optimal model to identify individuals at risk for falling out of care has the potential to improve retention. Our machine learning model was compared to logistic regression model and shown to have superior performance, be more adaptive, and have less disparate impact on minorities. Such a model will allow more precise prioritization of retention resources to patients likely to benefit most.

## Supplementary information

Supplementary Dataset 1.

## Data Availability

The datasets generated during and/or analyzed during the current study are not publicly available because they contain protected health information but are available from the corresponding author on reasonable request.
