# Supplementary material for: Predictive Analytics for Retention in Care in an Urban HIV Clinic
Source: Sci Rep. 2020 Apr 14;10:6421. doi: 10.1038/s41598-020-62729-x (PMC7156693; doi:10.1038/s41598-020-62729-x)
Supplement: Supplementary file 1 — Supplementary Dataset 1. [file 41598_2020_62729_MOESM1_ESM.docx]

**Predictive Analytics for Retention in Care in an Urban HIV Clinic**

Arthi Ramachandran PhD ^1,2,+^

Avishek Kumar PhD ^1,+^

Hannes Koenig MS ^1^

Adolfo De Unanue PhD ^1^

Christina Sung MBA ^1^

Joe Walsh PhD ^1^

John Schneider MD ^2^

Rayid Ghani MS ^1^

Jessica P. Ridgway MD, MS* ^2^

^1^ Center for Data Science and Public Policy, Department of Computer Science, University of Chicago, Chicago,

^2^ Chicago Center for HIV Elimination, Department of Medicine, University of Chicago, Chicago.

*Corresponding author: Jessica.ridgway@uchospitals.edu

+ These authors contributed equally to this work

**APPENDIX**

**eFigure 1 Temporal Cross Validation on EMR Dataset** Using temporal cross validation results in the 11 sets of model building (red) and validation datasets (blue). The solid lines indicate the time periods of the appointments included in the model building or validation datasets. The dotted lines represent the lag in time needed to calculate the outcome e.g., for a prediction made for an appointment on Feb 1, 2009, we need to consider the time period of Feb 1, 2009 - Aug 1, 2009 in order to compute the outcome of failure to access care in 6 months. For the outcome of failure to be retained in care, this lag is for 12 months, i.e. from Feb 1, 2009 – Feb 1, 2010. This lag is needed to ensure that no data that is used for validation is included in the model building. At any point in time, we use the maximum available data for model building. Models are then selected using the performance across all the validation data sets


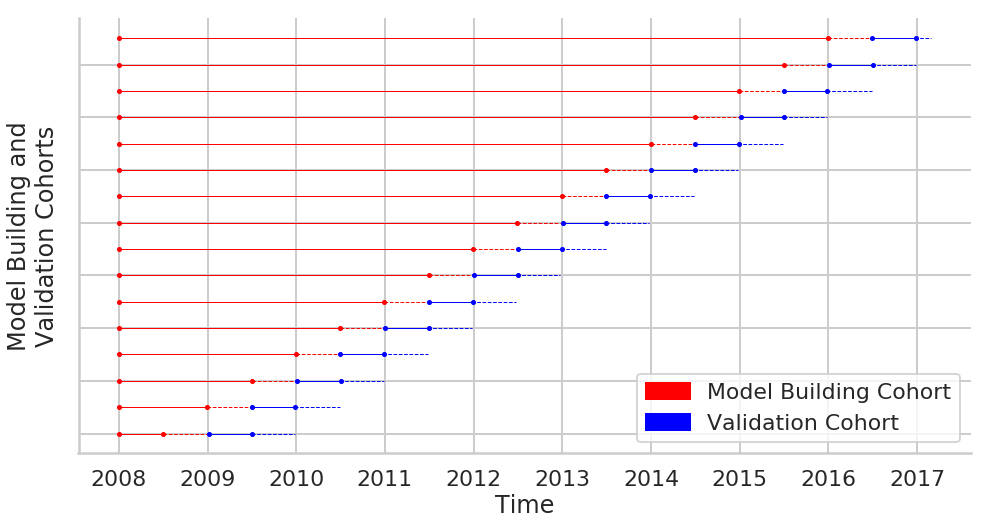


**eFigure 2 Fraction of patients (top, dotted) and appointments (bottom, solid) with no follow-up:** At the UCM HIV clinic, approximately 25% of the patients have no follow-up (gray, dotted) under the HRSA HAB definition of retention to care and approximately 10% of patients with no appointment in the following six months (orange, dotted). Interventions can potentially take place at the time of a patient’s appointment, casting the problem of retention as a point-of-service machine learning problem. Approximately 10% of appointments have no follow-up (gray and orange, solid) under either definition.


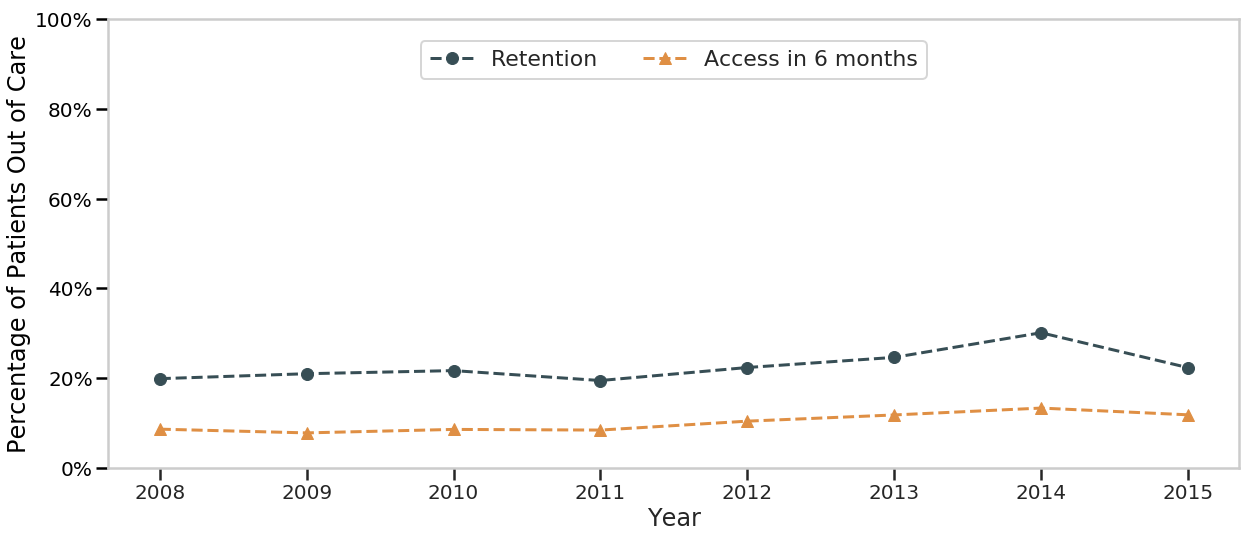

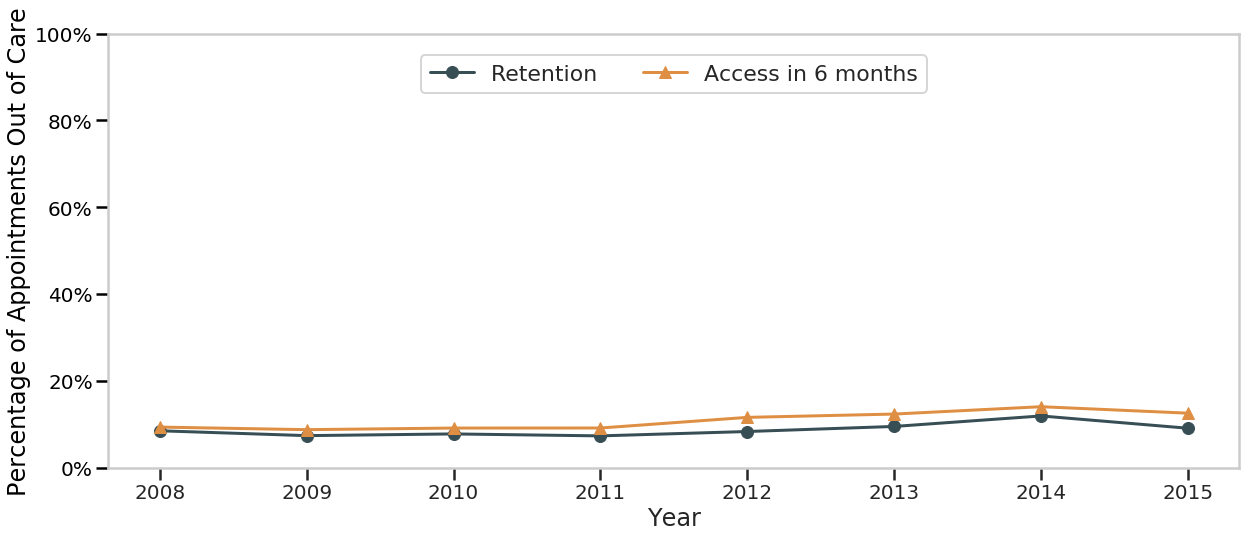


**
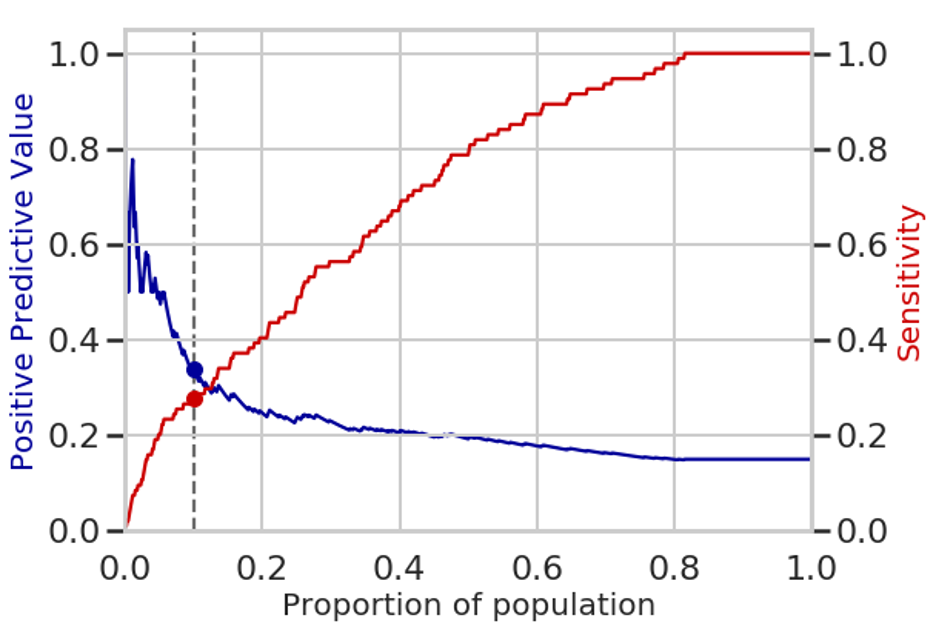
**

**
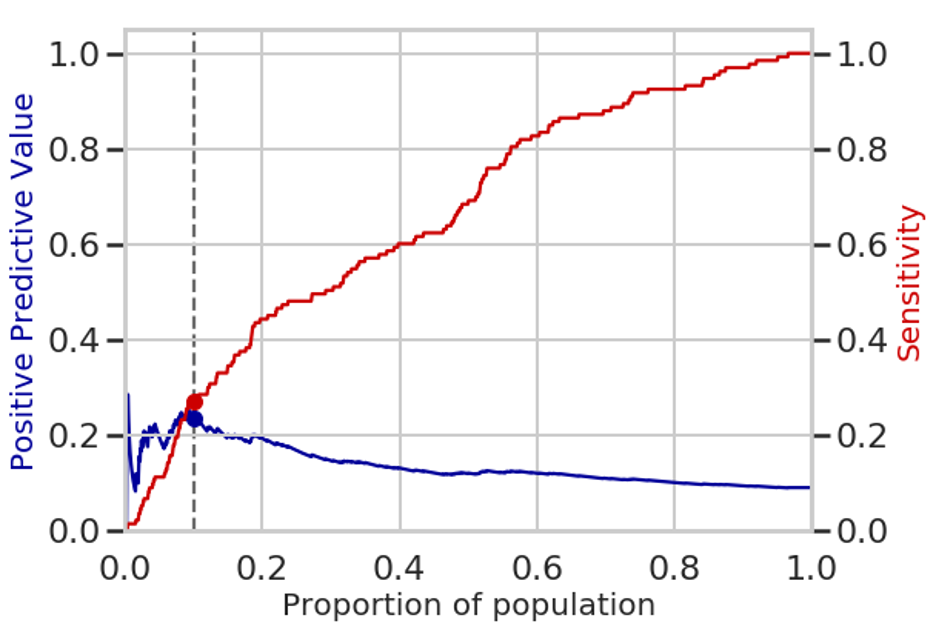
**

**eFigure 3 PPV vs Sensitivity for Retention in Care (Left) and Accessing Care (Right)**: This curve (also called the Precision-Recall curve) shows the PPV (blue) and sensitivity (red) for different threshold choices (x-axis). Model selection was optimized for best performance with a threshold of 10% of the population (circles, grey dotted line). The PPV and sensitivity are shown for both the retention in care model (left) and access to care model (right). Increasing the proportion of the population intervened on (x-axis) results in better sensitivity (red) but with a lower positive predictive value (blue). Note, we do not show the area under the curve since it captures the aggregate overall performance across every threshold.

**eTable 1 Predictor variables used in the model and the imputation method used when the data was missing**

| Predictor | Time Period | Imputation Value for Missing Data |
| --- | --- | --- |
| American Census Survey - derived |  |  |
| Fraction of population that is black in zipcode |  | 0 |
| Fraction of population that is native-born in zipcode |  | 0 |
| Fraction of population who have lived in the same house |  |  |
| for 5 years in zipcode |  | 0 |
| Fraction of population on SNAP in zipcode |  | 0 |
| Number of vehicles taken to work in zipcode |  | 0 |
| Fraction of population whose highest education is |  |  |
| high school in zipcode |  | 0 |
| CD4 Count |  |  |
| Has the patient been diagnosed with AIDS | in the last 6 months? | No |
|  | in the last year? | No |
|  | in the last 3 years? | No |
|  | ever? | No |
| Has the patient had a CD4 value between 200 and 500 | in the last 6 months? | Yes |
|  | in the last year? | Yes |
|  | in the last 3 years? | Yes |
|  | ever? | Yes |
| Has the patient had a CD4 value greater than 500 | in the last 6 months? | No |
|  | in the last year? | No |
|  | in the last 3 years? | No |
|  | ever? | No |
| Is the CD4/CD8 ratio less than 1 | in the last 6 months? | No |
|  | in the last year? | No |
|  | in the last 3 years? | No |
|  | ever? | No |
| Is the CD4/CD8 ratio between 1 and 2 | in the last 6 months? | No |
|  | in the last year? | No |
|  | in the last 3 years? | No |
|  | ever? | No |
| Is the CD4/CD8 ratio greater than 2 | in the last 6 months? | No |
|  | in the last year? | No |
|  | in the last 3 years? | No |
|  | ever? | No |
| Crime Statistics |  |  |
| Total number of reported crimes in zipcode | in last year | 0 |
| number of thefts in zipcode | in last year | 0 |
| Number of narcotics arrests in zipcode | in last year | 0 |
| Number of domestic violence incidents in zipcode | in last year | 0 |
| Demographics |  |  |
| Age |  | mean of cohort |
| Race |  | None |
| Ethnicity |  | None |
| Gender |  | None |
| Location (Zipcode) |  | None |
| Diagnoses |  |  |
| Number of psychiatric diagnoses | in last 6 months | 0 |
|  | in last year | 0 |
|  | in last 3 years | 0 |
|  | ever | 0 |
| Existence of psychiatric diagnoses | in last 6 months | No |
|  | in last year | No |
|  | in last 3 years | No |
|  | ever | No |
| Number of opportunistic infections | in last 6 months | 0 |
|  | in last year | 0 |
|  | in last 3 years | 0 |
|  | ever | 0 |
| Existence of opportunistic infections | in last 6 months | No |
|  | in last year | No |
|  | in last 3 years | No |
|  | ever | No |
| Number of sexually transmitted infections | in last 6 months | 0 |
|  | in last year | 0 |
|  | in last 3 years | 0 |
|  | ever | 0 |
| Existence of sexually transmitted infections | in last 6 months | No |
|  | in last year | No |
|  | in last 3 years | No |
|  | ever | No |
| Number of substance abuse diagnoses | in last 6 months | 0 |
|  | in last year | 0 |
|  | in last 3 years | 0 |
|  | ever | 0 |
| Existence of substance abuse diagnoses | in last 6 months | No |
|  | in last year | No |
|  | in last 3 years | No |
|  | ever | No |
| Number of positive toxicology screens | in last 6 months | 0 |
|  | in last year | 0 |
|  | in last 3 years | 0 |
|  | ever | 0 |
| Existence of positive toxicology screens | in last 6 months | No |
|  | in last year | No |
|  | in last 3 years | No |
|  | ever | No |
| Diagnosis category | in last 6 months | None |
|  | in last year | None |
|  | in last 3 years | None |
|  | ever | None |
| Number of days since first appointment |  | 0 |
| Hospitalizations |  |  |
| Number of hospital stays | in last 6 months | 0 |
|  | in last year | 0 |
|  | in last 3 years | 0 |
|  | ever | 0 |
| Number of times Infectious Disease provider seen | in last 6 months | 0 |
|  | in last year | 0 |
|  | in last 3 years | 0 |
|  | ever | 0 |
| Insurance |  |  |
| Number of different insurances | ever | 0 |
| Most common insurance | ever | None |
| Medications |  |  |
| Number of different medications | in last 6 months | 0 |
|  | in last year | 0 |
|  | in last 3 years | 0 |
|  | ever | 0 |
| Number of different ART medications | in last 6 months | 0 |
|  | in last year | 0 |
|  | in last 3 years | 0 |
|  | ever | 0 |
| Number of different ART medications |  |  |
| (deduplicated for generics) | in last 6 months | 0 |
|  | in last year | 0 |
|  | in last 3 years | 0 |
|  | ever | 0 |
| Number of different opioid medications | in last 6 months | 0 |
|  | in last year | 0 |
|  | in last 3 years | 0 |
|  | ever | 0 |
| Number of different psychiatric medications | in last 6 months | 0 |
|  | in last year | 0 |
|  | in last 3 years | 0 |
|  | ever | 0 |
| Number of different medications for opportunistic infections | in last 6 months | 0 |
|  | in last year | 0 |
|  | in last 3 years | 0 |
|  | ever | 0 |
| Previous Infectious Disease Appointment History |  |  |
| Number of completed ID appointments | in last 6 months | 0 |
|  | in last year | 0 |
|  | in last 3 years | 0 |
|  | ever | 0 |
| Number of cancelled ID appointments | in last 6 months | 0 |
|  | in last year | 0 |
|  | in last 3 years | 0 |
|  | ever | 0 |
| Number of no show ID appointments | in last 6 months | 0 |
|  | in last year | 0 |
|  | in last 3 years | 0 |
|  | ever | 0 |
| Average number of days between ID appointments | in last 6 months | 10 years |
|  | in last year | 10 years |
|  | in last 3 years | 10 years |
|  | ever | 10 years |
| Min number of days between ID appointments | in last 6 months | 10 years |
|  | in last year | 10 years |
|  | in last 3 years | 10 years |
|  | ever | 10 years |
| Max number of days between ID appointments | in last 6 months | 10 years |
|  | in last year | 10 years |
|  | in last 3 years | 10 years |
|  | ever | 10 years |
| Previous non-Infectious Disease Appointment History |  |  |
| Number of completed appointments | in last 6 months | 0 |
|  | in last year | 0 |
|  | in last 3 years | 0 |
|  | ever | 0 |
| Number of cancelled appointments | in last 6 months | 0 |
|  | in last year | 0 |
|  | in last 3 years | 0 |
|  | ever | 0 |
| Number of no show appointments | in last 6 months | 0 |
|  | in last year | 0 |
|  | in last 3 years | 0 |
|  | ever | 0 |
| Average number of days between appointments | in last 6 months | 10 years |
|  | in last year | 10 years |
|  | in last 3 years | 10 years |
|  | ever | 10 years |
| Min number of days between appointments | in last 6 months | 10 years |
|  | in last year | 10 years |
|  | in last 3 years | 10 years |
|  | ever | 10 years |
| Max number of days between appointments | in last 6 months | 10 years |
|  | in last year | 10 years |
|  | in last 3 years | 10 years |
|  | ever | 10 years |
| Infectious Disease Provider |  |  |
| Number of different ID providers | in last 6 months | None |
|  | in last year | None |
|  | in last 3 years | None |
|  | ever | None |
| Most common ID provider | in last 6 months | None |
|  | in last year | None |
|  | in last 3 years | None |
|  | ever | None |
| Retention History |  |  |
| Currently retained in care? |  | No |
| Total number of days retained |  | 0 |
| Total number of days unretained |  | 0 |
| Fraction of time retained |  | 0 |
| Fraction of time unretained |  | 0 |
| Number of changes in retention status |  | 0 |
| Number of drops in retention |  | 0 |
| Number of consecutive days retained |  | 0 |
| Number of consecutive days unretained |  | 0 |
| Number of days since last appointment |  | 0 |
| Viral Load |  |  |
| Is the patient virally supressed (<200) | in the last 6 months? | No |
|  | in the last year? | No |
|  | in the last 3 years? | No |
|  | ever? | No |
| Number of times the patient was virally supressed | in the last 6 months? | No |
|  | in the last year? | No |
|  | in the last 3 years? | No |
|  | ever? | No |
| Does the patient have a viral load |  |  |
| between 75 and 200 | in the last 6 months? | No |
|  | in the last year? | No |
|  | in the last 3 years? | No |
|  | ever? | No |
| Does the patient have a viral load |  |  |
| between 200 and 10,000 | in the last 6 months? | No |
|  | in the last year? | No |
|  | in the last 3 years? | No |
|  | ever? | No |
| Does the patient have a viral load |  |  |
| between 10,000 and 100,000 | in the last 6 months? | Yes |
|  | in the last year? | Yes |
|  | in the last 3 years? | Yes |
|  | ever? | Yes |
| Does the patient have a viral load |  |  |
| greater than 100,000 | in the last 6 months? | No |
|  | in the last year? | No |
|  | in the last 3 years? | No |
|  | ever? | No |
| What is the average magnitude change in viral load | in the last 6 months? | 0 |
|  | in the last year? | 0 |
|  | in the last 3 years? | 0 |
|  | ever? | 0 |
| What is the minimum magnitude change in viral load | in the last 6 months? | 0 |
|  | in the last year? | 0 |
|  | in the last 3 years? | 0 |
|  | ever? | 0 |
| What is the maximum magnitude change in viral load | in the last 6 months? | 0 |
|  | in the last year? | 0 |
|  | in the last 3 years? | 0 |
|  | ever? | 0 |
|  |  |  |
